# Anterior Hyaloid Fibrovascular Proliferation

**Published:** 2010-01

**Authors:** Bill Aylward, Ramin Tadayoni, Fernando Arevalo, Reza Karkhaneh

**Affiliations:** Consultant Vitreoretinal Surgeon and Medical Director, Moorfields Eye Hospital, London, UK; Department of Ophthalmology, Hôpital Lariboisière, AP-HP, Université Paris Diderot, Paris, France; Professor, University of Los Andes, Merida, Caracas, Venezuela; Professor, Vitreoretinal Service, Farabi Eye Hospital, Tehran University of Medical Sciences, Tehran, Iran

## CASE PRESENTATION

**Figure 1:**
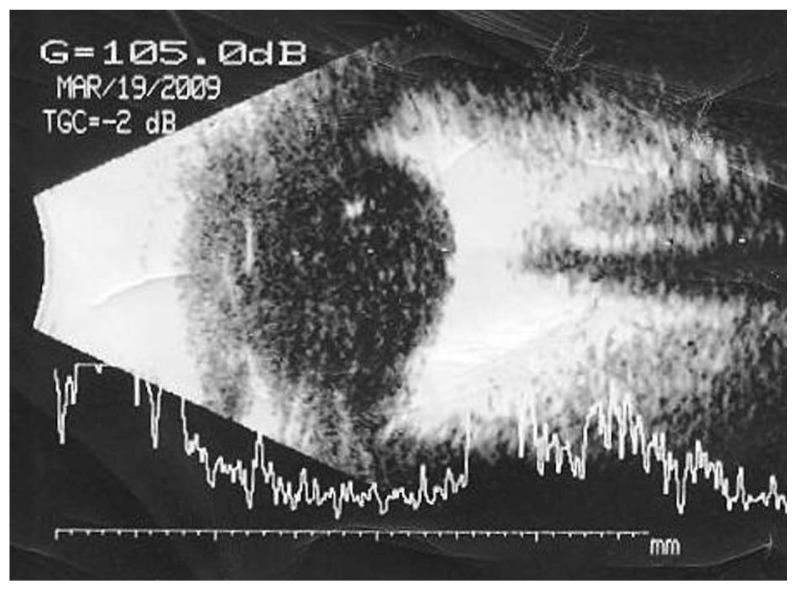
Vitreous hemorrhage one month following vitrectomy.

**Figure 2:**
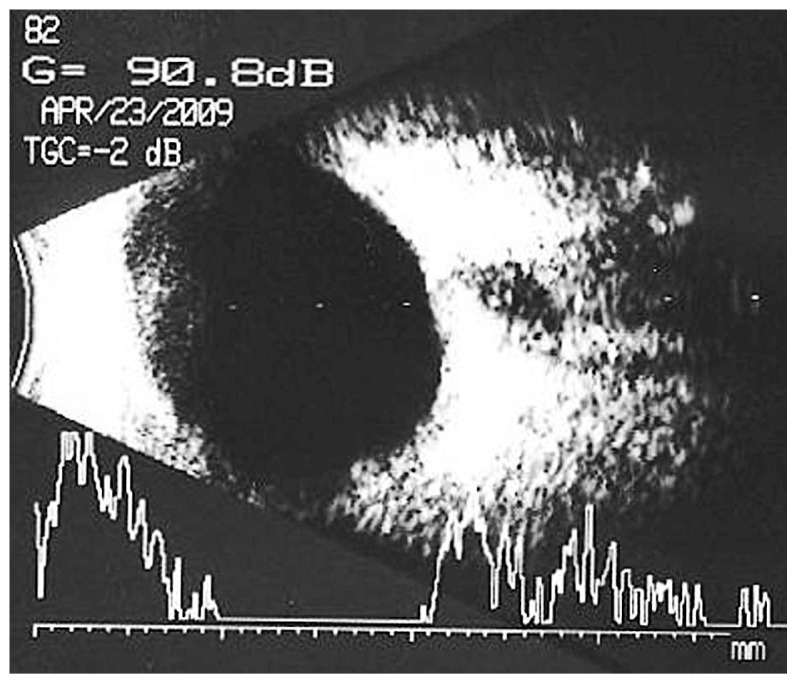
Non-clearing vitreous hemorrhage but attached retina three months after vitrectomy.

**Figure 3:**
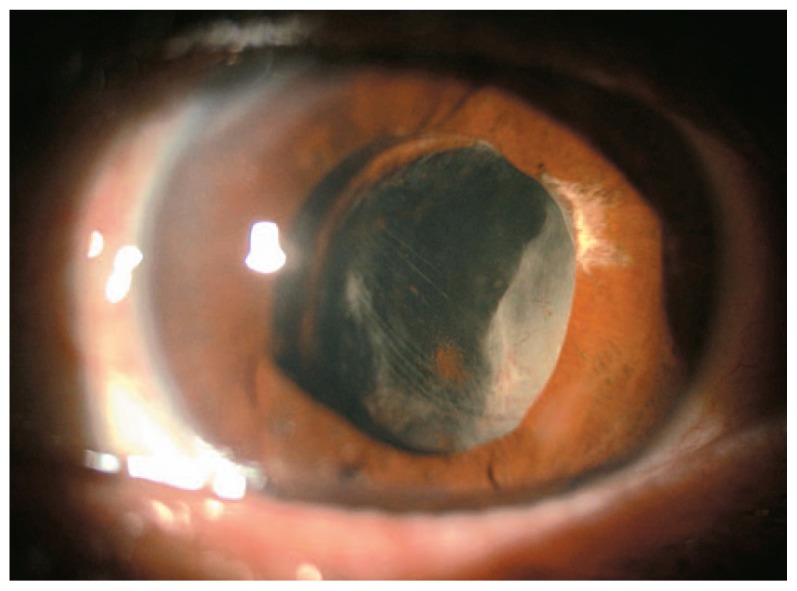
IOL capture and fibrovasular tissue posterior to the IOL ten months after cataract surgery.

**Figure 4:**
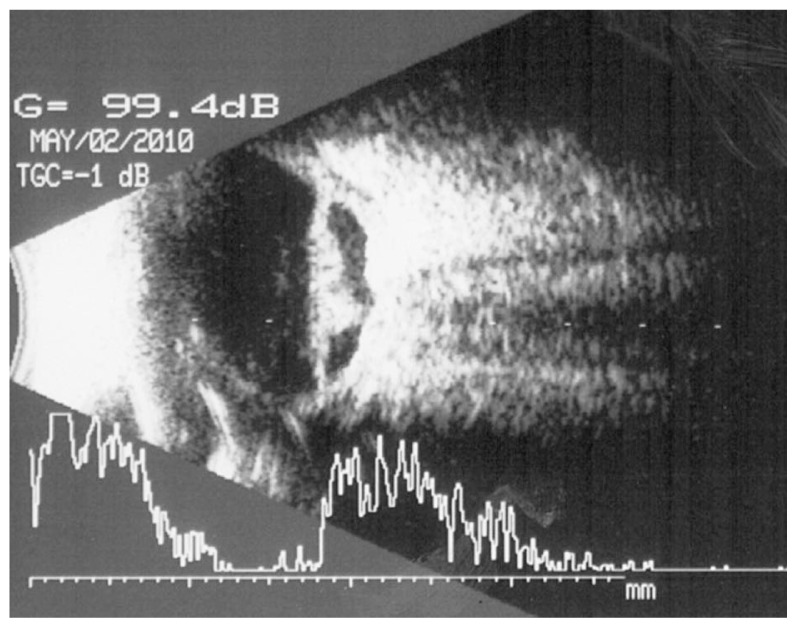
Posterior segment appearance 10 months after cataract surgery.

A 64-year-old diabetic lady underwent pars plana vitrectomy in her left eye for a taut posterior hyaloid face due to proliferative diabetic retinopathy (PDR). Visual acuity (VA) at baseline had been 20/120. She had previously received panretinal laser photocoagulation (PRP) and the retinopathy had been stable, but there was localized extrafoveal tractional retinal detachment in the inferonasal quadrant. After vitrectomy, she was discharged in good condition, VA of 20/400 and mild vitreous hemorrhage (VH). One month postoperatively, the density of the VH increased and VA decreased to counting fingers (CF) (Fig. 1). The VH was non-clearing for three months but on echography, the retina was attached (Fig. 2). VH density decreased one month later and the patient received additional peripheral laser therapy. Six months postoperatively, she underwent uncomplicated phacoemulsification with intraocular lens (IOL) implantation due to severe lens opacity. One month after cataract surgery, VA was 20/400, intraocular pressure (IOP) was 3 mmHg and there was fibrin deposition over the IOL. Fundus examination revealed regressed PDR. She received steroid

and cycloplegic drops and the condition remained stable three months after cataract surgery. On final examination, about 10 months after cataract surgery, VA deteriorated to hand motions, IOP was 5 mmHg, the IOL was partially captured by iris with fibrovasular tissue behind the IOL (Fig. 3); the fundus was not visible. The echography is shown in Figure 4.

Based on this presentation, what is your diagnosis, what treatment modality would you recommend and do you think there has been any pitfall in the management of this patient?

### Bill Aylward, FRCS, FRCOphth

This appears to be a case of anterior hyaloidal proliferation, which is a fibrovascular response of the vitreous base to anterior ischemia. There are a number of established risk factors including traction as an indication for surgery, recurrent hemorrhage, and cataract extraction.

Although it is stated that “fundus examination revealed regressed PDR” at six months, it is quite likely that the anterior retina remained very ischemic and continued to produce large quantities of vascular endothelial growth factor (VEGF) which diffused forward, aided by the state of pseudophakia. The low IOP is due to traction on the ciliary body. This is a very serious situation, and in retrospect might have been avoided by more aggressive trimming of the vitreous base at the time of initial surgery together with heavy peripheral panretinal photocoagulation.

If the patient is keen on further treatment now (and without it the eye is lost), I would recommend intravitreal injection of an anti-VEGF followed by further surgery 6 days later. This surgery should remove as much of the anterior fibrous tissue as possible, and apply additional PRP as far forward as possible. The surgeon will find a dense white band of fibrous tissue over the ciliary body, and I recommend that they resist the temptation to peel this off! Finally I recommend silicone oil tamponade, as this will help counter the hypotony, as well as acting as a ‘VEGF insulator’ to guard against furthers proliferation.

Suggested Readings1LewisHAbramsGWWilliamsGAAnterior hyaloidal fibrovascular proliferation after diabetic vitrectomyAm J Ophthalmol1987104607613244650110.1016/0002-9394(87)90173-52UlbigMRHykinPGFossAJSchwartzSDHamiltonPAAnterior hyaloidal fibrovascular proliferation after extracapsular cataract extraction in diabetic eyesAm J Ophthalmol1993115321326768018610.1016/s0002-9394(14)73582-2

### Ramin Tadayoni, MD

Based on the provided information, this lady’s left eye presents a diabetic anterior fibrovascular proliferation (Fig. 3) associated with severe tractional retinal detachment (Fig. 4). IOP is low and the eye seems already on the way to phthisis bulbi. At this stage and despite all heroic interventions, the prognosis remains very poor. A vitrectomy with dissection of proliferations and probably anterior retinectomy, IOL removal and silicone injection could be a last chance treatment.

These conditions could sometimes be prevented by early diagnosis and intervention: generous photocoagulation of all anterior ischemic areas, treatment of significant retinal detachment in particular in the anterior retina and intraocular use of anti-VEGF agents. This complication must be in mind when hypotony, especially associated with a vitreous hemorrhage, is diagnosed after any surgery for diabetic retinopathy.

Further upstream, the risk of these complications usually justifies a reoperation if the fundus remains inaccessible for more than one month after vitrectomy for diabetic retinopathy due to vitreous hemorrhage, to check the retina and treat the cause of bleeding. In high-risk cases (in particular cases with proliferation extending forward to the equator), some surgeons systematically inject anti-VEGF agents at the end of the vitrectomy, even though its benefit has not been clearly proven.

### Fernando Arevalo, MD

This is a 64-year-old woman with diabetes mellitus and history of decreased vision in the left eye, pars plana vitrectomy and PRP for management of PDR. Later on she underwent phacoemulsification with IOL implantation. Sixteen months after initial evaluation, VA was CF and IOP was 5 mmHg. Examination revealed that the IOL was partially captured, there was fibrovasular tissue behind the IOL, and the fundus was not visible. Echography demonstrated vitreous hemorrhage, tractional retinal detachment (TRD) with proliferation and a posterior closed funnel. The choroid is thickened, and the axial length of the eye seems decreased as compared to previous echographic images. It is clear that this is a case of a TRD, fibrinoid syndrome and a prephthisical eye following vitrectomy, PRP, and phacoemulsification with IOL implantation for management of severe PDR.

At this point, I would consider surgery if this is the only eye. My approach would be to perform a 23-gauge transconjunctival sutureless vitrectomy with membrane peeling, IOL removal and silicone oil (SO) injection. My preference on a complicated case like this would be to use 5,000 centistoke (cs) SO. We have previously reported a higher rate of complications using 1,000 cs SO for complex retinal detachment repair including retinal redetachments, reproliferation, glaucoma, hypotony, keratopathy, cataract; furthermore, SO emulsification tends to occur not only more frequently but also faster with 1000 cs SO. Effective management for postoperative hypotony is problematic, revision of vitrectomy with peeling of membranes from the ciliary processes might be of value. In addition, subconjunctival injections of long-acting (depot) steroids may be given (anterior subconjunctival injections as opposed to posterior sub-Tenon injections are more likely to produce an elevation/normalization of IOP). No intravitreal bevacizumab should be used at this stage as it could induce worsening of the TRD.

In retrospect, I would have treated the vitreous hemorrhage earlier with a fluid-air/ gas exchange to avoid fibrin development. In addition, I would have treated the fibrin deposition over the IOL with intracameral tissue plasminogen activator at a dose of 25 μg/0.1 mL. Any fibrin appearing in the frontal plane as a result of severe inflammation may result in development of a cyclitic membrane and phthisis bulbi in severe cases. Steroids do not appear to retard healing of any of the ocular structures and should be adequately used to suppress all inflammation.

In summary, this is a 64-year-old woman with diabetes mellitus and history of decreased vision in the left eye, pars plana vitrectomy and PRP for PDR, and phacoemulsification with IOL implantation. She developed a TRD, fibrinoid syndrome and a prephthisical eye with hypotony. Surgery is warranted if this is an only eye but the patient should be warned that the prognosis is poor.

Suggested Readings1CamachoHArevaloJFPeñarandaJSilicone oil removal in vitreoretinal surgeryRev Soc Colomb Oftalmol19951828322ArevaloJFCamachoHThe recent article by Shah et al titled “short-term outcomes of 25-gauge vitrectomy with silicone oil for repair of complicated retinal detachment” (Retina 2008;28:723–728)Retina2009294164171928729110.1097/IAE.0b013e3181921fb13ArevaloJFGarciaRAFernandezCFAnterior segment inflammation and hypotony after posterior segment surgeryOphthalmol Clin North Am2004175275371553374610.1016/j.ohc.2004.06.0044ArevaloJFMaiaMFlynnHJrSaraviaMAveryRLWuLTractional retinal detachment following intravitreal bevacizumab (Avastin) in patients with severe proliferative diabetic retinopathyBr J Ophthalmol2008922132161796510810.1136/bjo.2007.127142

### Reza Karkhaneh, MD

This is a case of vitreous hemorrhage after vitrectomy for PDR, a common complication with an incidence of 12% to 63%. The hemorrhage may appear within the first few weeks after surgery or months later. The source of early bleeding in such cases may be residual blood in the peripheral vitreous skirt, iatrogenic intraoperative injury to retinal vessels, and incomplete removal of fibrovascular tissues. The cause of late vitreous hemorrhage (one month postoperatively in this patient) can be fibrovascular proliferation from the sclerotomy sites or from the vitreous base. The vitreous hemorrhage persisted for three months after vitrectomy, during this period the retina was attached as revealed by B scan ultrasonography.

In this case it may be difficult to differentiate the origin of vitreous hemorrhage by conventional methods of examination using an indirect ophthalmoscope or B scan ultrasonography because of poor visibility and limited scope of evaluation of anterior segment structures to detect complications at the sclerotomy sites. On the other end, ultrasound biomicroscopy (UBM) is helpful to provides images of fibrovascular ingrowth at sclerotomy sites. Postvitrectomy vitreous hemorrhage clears more rapidly in aphakic eyes (at an average of 5.3 weeks) than in phakic eyes (at an average of 16.2 weeks).

Vitreous hemorrhage decreased four months after surgery in this case and the patient received additional peripheral laser therapy. Six months after vitrectomy the patient underwent uncomplicated phacoemulsification with IOL implantation. An important finding was ocular hypotonia one month after cataract surgery which may have been due to peripheral retinal detachment or anterior retinal displacement and ciliary body detachment secondary to fibrovascular proliferation from the sclerotomy sites or from the vitreous base. At this stage of the disease, UBM imaging is highly recommended. UBM also is helpful for early diagnosis and timely management of such complications.

Presence of any fibrovascular proliferation at the sclerotomy site would indicate the need for more aggressive retinal ablation, especially to the periphery, with the indirect laser delivery system, transconjunctival cryopexy, or transconjunctival diopexy with diode laser. It appears that these complications could have been diagnosed and treated earlier.

Finally about 10 months after cataract surgery there was massive fibrovasular tissue behind the IOL and IOP was 5 mmHg, the B-scan ultrasonography showed advanced TRD of the posterior and peripheral retina. These findings are consistent with anterior hyaloidal fibrovascular proliferation. It is a severe complication after vitrectomy for PDR, which, if not treated aggressively can lead to phthisis bulbi. Therefore despite the poor prognosis and visual outcome in this case, it is recommended to perform vitrectomy, remove all fibrovascular proliferation at the sclerotomy sites and peripheral retina, repair the retinal detachment, aggressively perform retinal ablation especially to the periphery of the retina with endolaser, indirect laser delivery system, cryopexy, or diopexy, and inject silicone oil. I would prefer to inject intravitreal bevacizumab and triamcinolone acetonide at the conclusion of surgery in this case.

Suggested Readings1YangCMYehPTYangCHIntravitreal long-acting gas in the prevention of early postoperative vitreous hemorrhage in diabetic vitrectomyOphthalmology20071147101727590810.1016/j.ophtha.2006.07.0472BhendeMAgraharamSGGopalLSumasriKSukumarBGeorgeJUltrasound biomicroscopy of sclerotomy sites after pars plana vitrectomy for diabetic vitreous hemorrhageOphthalmology2000107172917361096483710.1016/s0161-6420(00)00213-x3LandersMBPerrakiADManagement of post-vitrectomy persistent vitreous hemorrhage in pseudophakic eyesAm J Ophthalmol20031369899931464420710.1016/s0002-9394(03)00718-94HershbergerVSAugsburgerJJHutchinsRKRaymondLAKrugSFibrovascular ingrowth at sclerotomy sites in vitrectomized diabetic eyes with recurrent vitreous hemorrhage: ultrasound biomicroscopy findingsOphthalmology2004111121512211517797410.1016/j.ophtha.2003.08.043

## References

[1] Lewis H, Abrams GW, Williams GA (1987). Anterior hyaloidal fibrovascular proliferation after diabetic vitrectomy. Am J Ophthalmol.

[2] Ulbig MR, Hykin PG, Foss AJ, Schwartz SD, Hamilton PA (1993). Anterior hyaloidal fibrovascular proliferation after extracapsular cataract extraction in diabetic eyes. Am J Ophthalmol.

